# COVID-19 postvaccination adverse events and vaccine hesitancy among hospital employees: Is there a link?

**DOI:** 10.1017/ash.2022.134

**Published:** 2022-05-16

**Authors:** Anastasiia Weiland, Rangit Vallapureddy, Sarah Boaitey, Keyvan Ravakhah

## Abstract

**Background:** Vaccination against COVID-19 has demonstrated high efficacy in preventing illness severe enough to result in hospitalization. Despite these data, universal vaccine adoption by different population groups, including hospital employees, has been a challenging public health task. Vaccine-associated adverse events, the novelty of the vaccines, and the absence of long-term follow-up data have been reported as major contributors to COVID-19 vaccines mistrust. We sought to quantify postvaccination adverse events, to assess their correlation with unvaccinated status, and to evaluate other factors contributing to COVID-19 vaccination hesitancy. **Methods:** In a 240-bed community hospital located in a metropolitan area in the United States, we conducted a voluntary and anonymous online survey among contracted employees between September and November 2021. The study protocol was approved by the institutional review board at our facility. **Results:** Of all 185 responders, 143 (77%) were female, 95 (51%) were aged <51 years, and 146 were White (79%). Most (n = 100, 54%) reported no past medical history. Most common comorbidities included heart disease (n = 45, 24%), diabetes (n = 20, 11%), and chronic lung disease (n = 17, 9%). Among those surveyed, 178 were vaccinated either fully (n = 172, 93%) or partially (n = 6, 3%), and 7 (4%) were unvaccinated. Moderna was the most common vaccine received (n = 152, 85%). Those who received a 2-dose series reported experiencing more adverse events after the second dose than after the first dose (710 vs 451) of either Moderna or Pfizer vaccine. Adverse events included pain at the injection site (n = 257, 22%), fatigue (n = 178, 15%), chills (n = 133, 11%), muscle pain (n = 120, 10%), and headache (n = 117, 10%). Also, 2 responders reported omitting the second dose due to the severity of symptoms after the first dose of both Moderna and Pfizer vaccines. Concern for safety (n = 5, 71%) was the leading reason for vaccine refusal among unvaccinated followed by concern for efficacy (n = 3, 43%), lack of trust in government promoting vaccination (n = 3, 43%), religious reasons (n = 2, 28%), and immunity due to prior COVID-19 (n = 2, 28%). In addition, 3 responders reported intent to be vaccinated in the future. **Conclusions:** Most of the responders reported at least 1 adverse event related to COVID-19 vaccination. No severe adverse events were reported; however, a high prevalence of self-limited postvaccination adverse events might be misinterpreted as a concern for vaccine safety, as seen among surveyed unvaccinated individuals in our cohort. Targeted education is needed to limit knowledge gaps and address existing cognitive biases in COVID-19 vaccination among hospital employees.

**Funding:** None

**Disclosures:** None

